# Country-level and regional COVID-19 burden and determinants across OECD member states and partner countries

**DOI:** 10.1265/ehpm.22-00054

**Published:** 2022-10-21

**Authors:** Nlandu Roger Ngatu, Kazuto Tayama, Kanae Kanda, Tomohiro Hirao

**Affiliations:** Department of Public Health, Kagawa University Faculty of Medicine, Kagawa, Japan

**Keywords:** Case fatality rate, Coronavirus disease 2019, Determinant, Years of life lost

## Abstract

**Background:**

COVID-19 pandemic is tremendously impacted by socioeconomic and health determinants worldwide. This study aimed to determine factors associated with COVID-19 fatality among member states and partner countries of the Organization for Economic Cooperation and Development (OECD).

**Methods:**

An ecological study was conducted using COVID-19 data of 48 countries for the period between 31 December 2019–31 December 2021. The outcome variables were COVID-19 case fatality rate (CFR) and years of life lost to COVID-19 (YLLs). Countries’ sociodemographics and COVID-19-related data were extracted from OECD website, Our World in Data, John Hopkins Coronavirus Resource Center, Economist Intelligence Unit (EIU) and WHO.

**Results:**

In the first year of the pandemic (December 2019–January 2021), highest CFR was observed in Mexico, 8.51%, followed by China, 5.17% and Bulgaria, 4.12%), and highest YLLs was observed in Mexico, 2,055 per 100,000. At regional level, highest CFR was observed in North & central America, 4.25 (3.71) %, followed by South America (2.5 (0.1) %); whereas highest YLLs was observed in South America region 1457.5 (274.8) per 100,000, followed by North & central America, 1207.3 (908.1) per 100,000. As of 31 December 2021, Mexico (7.52%) and Bulgaria (4.78%) had highest CFR; on the other hand, highest YLLs was observed in England, 26.5 per 1,000, followed by the United States, 25.9 per 1,000. At regional level, highest CFR (3.37(3.19) %) and YLLs (16.7 (13) per 1,000) were both observed in North & central America. Globally, the analysis of the 2-year cumulative data showed inverse correlation between CFR and nurse per 10,000 (R = −0.48; p < 0.05) and GDP *per capita* (R = −0.54; p < 0.001), whereas positive correlation was observed between YLLs and elderly population rate (R = 0.66; p < 0.05) and overweight/obese population rates (R = 0.55; p < 0.05).

**Conclusion:**

This study provides insights on COVID-19 burden among OECD states and partner countries. GDP *per capita*, overweight/obesity and the rate of elderly population emerged as major social and health determinants of COVID-19 related burden and fatality. Findings suggest that a robust economy and interventions designed to promote healthy longevity and prevent weight gain in at-risk individuals might reduce COVID-19 burden and fatality among OECD states and partner countries.

**Supplementary information:**

The online version contains supplementary material available at https://doi.org/10.1265/ehpm.22-00054.

## 1. Introduction

Severe acute respiratory syndrome coronavirus 2 (SARS-CoV-2) is the causal pathogen of the coronavirus disease 2019 (COVID-19) [1], which can result in a hyperinflammatory state and a diversity of complications [2]. The pandemic has tremendously impacted socioeconomic, environmental and healthcare determinants of health in most countries of the world, resulting in job and income losses, social networks disruption, reduced mobility, increased suicide and crime rates, and changes of patterns of common epidemic or endemic communicable (CDs) and noncommunicable diseases (NCDs) [3, 4]. The SARS-CoV-2 infection outbreak originated at the Chinese city of Wuhan where a cluster of pneumonia cases of unknown origin was reported by health officials on 31 December 2019 [5, 6]. Since, the disease has continued to spread all over the world, overwhelmingly surpassing its predecessors, namely Severe acute respiratory syndrome coronavirus 1 (SARS-CoV1) and Middle East respiratory syndrome coronavirus (MERS-CoV), in terms of morbidity and mortality, as well as the spatial range of epidemic areas. At the end of January 2020, the World Health Organization (WHO) declared a Public Health Emergency of international concern [7–9].

COVID-19 has already affected 110.7 million cases globally and over 2.4 million deaths reported since its declaration as a pandemic, as of 23 February 2021 [10]. Recent epidemiological data show that countries of the Organization for Economic Cooperation and Development (OECD) and OECD invited partner countries are those that bear the heaviest COVID-19 burden in the world, in terms of disease morbidity and mortality. Region of the Americas were the most affected area in the world, with 49,296,115 cumulative cases (45%) and 1,171,294 cumulative deaths (48%), whereas Western Pacific and Africa Regions accounted for less than 5% for both indicators [10]. Meanwhile, these numbers increased tremendously: 532,201,219 confirmed cases globally (222,417,177 cases in Europe; 158,983,746 cases in the Americas; 61,735,224 cases in western Pacific; 58,217,287 cases in South-East Asia, 21,807,376 cases in Eastern Mediterranean; 9,039,645 cases in Africa) and 6,305,358 deaths, as of 10 June 2022 [11]. This disparity in the regional distribution of SARS-CoV-2 infection burden suggests the possibility of the existence of disease determinants that might amplify the disease spread in some parts of the world.

Previous studies have shown, for example, that socioeconomic disparities affect differently communities and countries in terms of COVID-19 morbidity and mortality [12, 13]. Investigating the impact of sociodemographics and health indicators in COVID-19 burden is an important public health step towards understanding why the pandemic disproportionately affects countries across the globe. It helps to design efficient evidence-base interventions to address the health crisis [14].

To our knowledge, there have been no studies that thoroughly searched to explore country and regional level factors that influence COVID-19 fatality in the group of OECD members states. Thus, the objective of this study was to determine the health, sociodemographic, economic and political factors associated with COVID-19 fatality in 48 OECD member states and invited/partner countries. The study hypothesis was that countries and the region with highest elderly population would have experienced the most severe COVID-19 outcomes.

## 2. Materials and methods

### 2.1. Study design and data sources

This was an ecological study that used recent COVID-19 data of 48 countries for the period between December 2019 through 31 December 2021. The 48 countries included 37 OECD member states (Austria, Australia, Belgium, Canada, Chile, Colombia, Czech Republic, Denmark, Estonia, Finland, France, Germany, Greece, Hungary, Iceland, Ireland, Israel, Italy, Japan, Korean, Latvia, Lithuania, Luxembourg, Mexico, The Netherlands, New Zealand, Norway, Poland, Portugal, Slovak Republic, Slovenia, Spain, Sweden, Switzerland, Turkey, the United Kingdom and the United States of America) and 11 OECD partner countries (Argentina, Brazil, Bulgaria, China, India, Indonesia, Romania, Russia, Saudi Arabia, South Africa) plus Taiwan.

Data used in this study originated from four sources: OECD website, Our World in Data, Johns Hopkins Coronavirus Resource Center, Economist Intelligence Unit (EIU), WHO and the World Bank. They are anonymous and freely accessible.

### 2.2. Covariates, outcome variables and ethical consideration

Health indicators were (1) Cumulative COVID-19 cases, (2) COVID-19 case fatality rate (CFR) and (3) years of life lost to COVID-19 (YLLs). Countries’ sociodemographic, economic and political characteristics were used as potential predictors of outcome variables: population, elderly population rate (65 years of age or older), literacy rate, higher education completion rate, gross domestic index (GDP) *per capita* and democracy index (Table 1). In this study, only CFR and YLLs were considered outcome variables.

➢ COVID-19 case was defined according WHO: an individual who meets clinical criteria and epidemiological criteria, a patient with severe acute respiratory illness, or an asymptomatic person not meeting epidemiologic criteria with a positive SARS-CoV-2 test, as described previously [15].
➢ Democracy index is calculated based on 60 indicators grouped in 5 categories; it measures pluralism, civil liberties, country’s political culture and varies from 0 to 10. In addition to a numeric score, it categorizes each country into one of the political regime types: full democracy, flawed democracy, hybrid regimes and authoritarian regimes [16].
➢ CFR is an indicator that estimates the proportion of people who die from a specific disease among all subjects diagnosed with the disease over a period of time. We calculated CFR for each country using the following formula:
$$\text{CFR (\%)}=\frac{\text{Number of COVID-19 deaths}\times 100}{\text{Number of COVID-19 cases}}$$
➢ According WHO, YLLs is a measure of premature death; it is calculated from the number of deaths from a disease multiplied by a global standard life expectancy at the age of death occurrence. YLLs data used in the study are from COVID-19 databases; CFRs were calculated by the authors.

To conduct this study, informed consent was not required given that our data sources were anonymous and freely accessible.

**Table 1:** List of outcome and explanatory variables

**Category**	**Variables**	**Sources of data**
Health indicators	- Cumulative COVID-19 cases - Cumulative COVID-19 deaths - Years of life lost due to COVID-19 (YLLs)	WHO website (Feb 2021) [17]; OECD website (Feb 2021) [18]; John Hopkins COVID-19 Resource Center [19]; European Review for Medical and Pharmaceutical Sciences [20]; WHO website (10 June 2022) [11] Our World in Data (13 June 2022) [21]
Health system indicators	- Number of doctors per 10,000 inhabitants - Number of nurses per 10,000 inhabitants	WHO website (Feb 2021) [17] OECD website (Feb 2021) [18]
Sociodemographic, economic, political characteristics	- Population; elderly population rate (65 years of age or older) - Higher education completion rate - Country’s democracy index - Gross domestic product (GDP) *per capita*	OECD website (Feb 2021) [18] Our World in Data (Feb 2021) [22] The Economist Intelligence Unit [16] OECD stat (29 May 2022) [23]

### 2.3. Statistical analysis

CFR and YLLs are used as continuous data; they are pre sented as means and standard deviations (SD) or standard errors (SE) with their corresponding 95% confidence intervals (CIs). Regional comparisons of CFR and YLLs were performed using the one-way analysis of variance (ANOVA). Normality test (Shapiro-Wilk test) was performed for both outcome variables (CFR, YLLs).

To explore the relationship between each outcome variables (CFR, YLL) and the covariates or explanatory variables (GDP, high education completion rate, elderly population rate, overweight/obese population rate, etc.), bi variate linear regression analysis was used, with adjustment for country’s elderly population rate, for data related to the first year of the pandemic, given that those data were found to be not normally distributed. In order to use the regression analysis, multicollinearity between the covariates was checked using variance inflation factor (VIF). On the other hand, Spearman correlation test was employed for overall data (as of 31 December 2021) given that outcome variables data were found to be normally distributed.

Statistical analyses were performed with the use of Stata statistical software (Stata Corp., TX, USA), whereas graphical images were built using JMP software version 15 (SAS, Cary, NC, USA). Statistical significance was set at p-value (double sided) less than 0.05.

## 3. Results

### 3.1. General COVID-19 epidemic features in 48 OECD member and partner countries (2019–2021)

During the first year of the pandemic (December 2019–January 2021), mean cumulative cases was 1,819,206 (598,375.6) (Table 2); countries with highest cumulative COVID-19 cases (as of January 2020) were from North & central America (USA: 26,069,046; Mexico: 1,857,230), Western Europe (England: 3,796,088 cases; France: 3,167,274 cases; Spain: 2,743,119 cases; Germany: 2,224,911 cases); Asia-Pacific region (India: 10,746,174 cases; Indonesia: 1,066,313 cases); South America (Brazil: 9,176,975 cases; Colombia: 2,086,806 cases) (not shown). Cumulative COVID-19 deaths were 39,388.7 (10,878.8) (Table 2), with the United States (439,439 deaths), Brazil (223,945 deaths) and Italy (88,279 deaths) having highest cumulative deaths (not shown).

**Table 2:** Sociodemographics, health/health system indicators of OECD states and partner countries (Dec. 2019–31 Dec. 2021)

**Variables**	**Mean (SD)**	**95% CI**
***1. Sociodemographics & health system indicators***		
Population (×10,000)	10361.6 (28185.3)	21774–18545.7
Rate of elderly population (65 y. or older) (%; n = 48)	16.00 (0.79)	14.41–17.58
Higher education completion rate (%; n = 48)	47.4 (2.06)	3.22–49.18
Proportion of overweight & obese pop. (%) (n = 48)	60.07 (3.69)	52.09–68.07
Number of doctors (per 10,000) (n = 46)	3.26 (.84)	1.31–5.27
Number of nurses (per 10,000) (n = 46)	8.18 (3.96)	3.04–18.9
Democracy index (n = 48)	7.55 (0.24)	7.07–8.03
GDP *per capita* in 2020 (GDP1) (US$; n = 48)	33,598.87 (3,528.0)	26,501.5–40,696.3
GDP per capita in 2021 (GDP2) (US$; n = 48)	49,686 (3,882)	41,820.3–57,551.7
***2. Health indicators***		
Cumulative COVID-19 cases in 2020 (Total number; n = 48)	1,819,206 (598,375.6)	615,429.7–3,022,983
Cumulative COVID-19 cases/million in 2021 (n = 48)	113,766 (9,533.8)	94,551.8–132,980.2
Cumulative COVID-19 deaths in 2020 (Total number; n = 48)	39,388.7 (10,878.8)	17,503.4–61,274.2
Cumulative COVID-19 deaths/million in 2021 (n = 48)	1,655.4 (161.9)	1,329.1–1,981.8
COVID-19 case fatality rate in 2020 (**CFR1**) (%; n = 48)	2.25 (0.2)	1.88–2.64
COVID-19 case fatality rate in 2021 (CFR2) (%; n = 48)	1.63 (0.19)	1.23–2.03
Years of life lost to COVID-19 per 100,000 (**YLL1**) in 2020 **(n = 48)**	550.86 (84.97)	379.51–722.21
Years of life lost to COVID-19 per 1000 in 2021 (YLL2) (n = 48)	11.31 (1.76)	7.68–14.95

*Notes: SE, standard error; CI, confidence interval; CFR, case fatality rate.

As of 31 December 2021, overall cumulative COVID-19 cases (for the entire 2-year study period) was 113,766 (9,533.8) per million (Table 2); Slovakia (251,608 cases per million), Czech Republic (230,847 cases per million), Slovenia (223,237 cases per million), and England (190,078 cases per million) had highest number of infected individuals (Table 2). Overall there were 1,655.4 (161.9) deaths/million, and countries with high COVID-19 fatality were Bulgaria (4,488 deaths/million) and Hungary (4,067 deaths/million).

In the first year of the pandemic, overall CFR1 was 2.25% (Mexico, 8.51%; China, 5.17%; Bulgaria, 4.12%; Greece, 3.69%; Italy, 3.47%) (Table 2). Overall YLL1 was 550.86 (84.97) per 100,000 (Table 2), with Mexico (2,055), Columbia (1,773) and Brazil (1,593) being countries that bore the heaviest burden in terms of years of life lost. On the other hand, when considering the 2-year cumulative data as of 31 December 2021, CFR2 decreased slightly (1.63%) with Mexico (7.52%), Bulgaria (4.78%), Indonesia (3.38%) and Rumania (3.25%) having high rates. Overall YLL2 was 11.31 (1.76) per 1,000, and England (26.5 per 1000), the United States (25.9 per 1000) and Rumania (25.4 per 1000) were countries with highest years of life lost (Table 2).

**Fig. 1:**
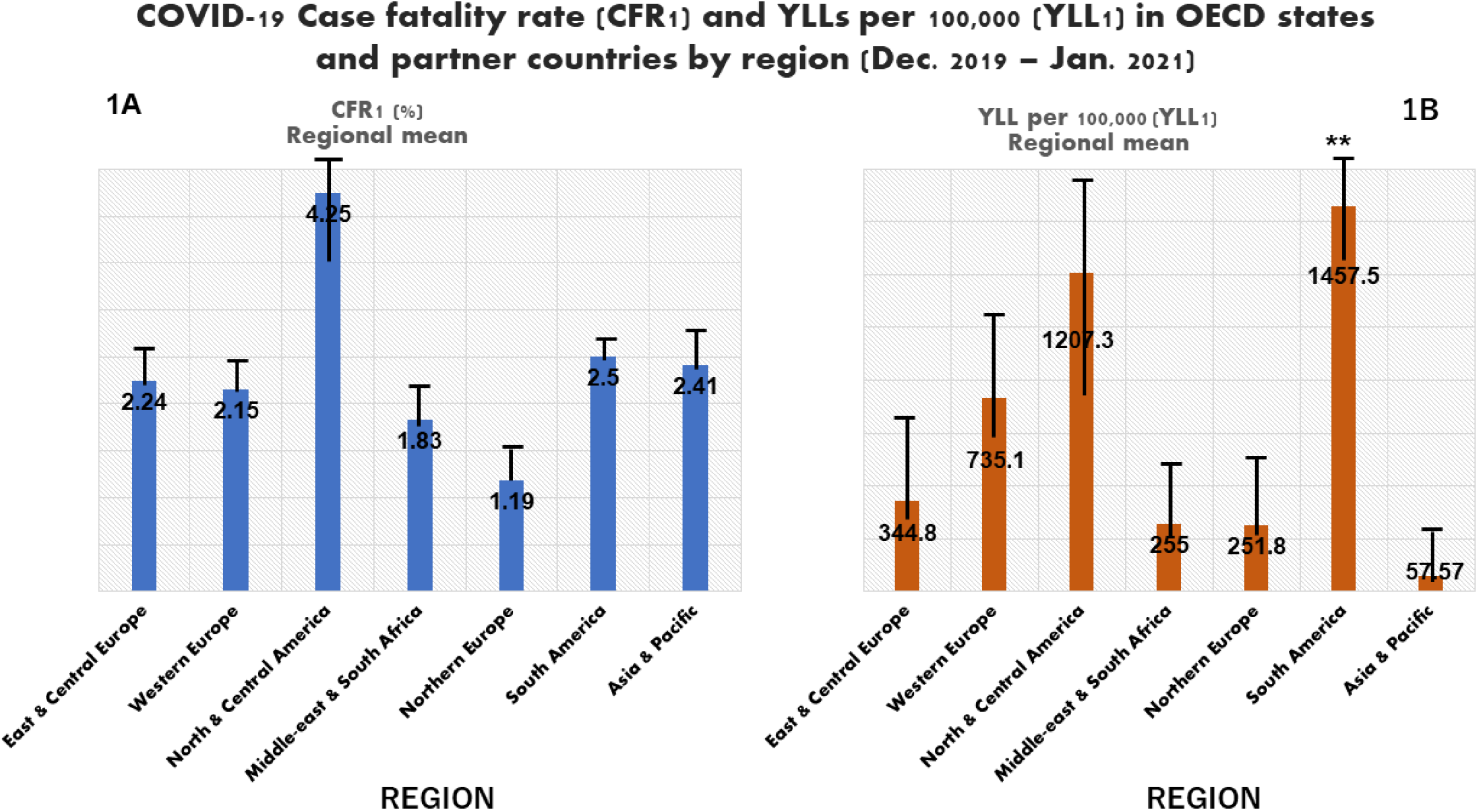
COVID-19 CFR (**A**) and YLL (**B**) in OECD states/partner countries by region (Dec. 2019–Jan. 2021). OECD, Organization for Economic Co-operation and Development; CFR1, case fatality rate in the first year of the pandemic; YLL1, years of life lost due to COVID-19 in the 2-year study period; **, p-value less than 0.001. The figure 1A–B shows a higher CFR in north & central America region, and higher YLL in South America region in the first year of COVID-19 pandemic.

**Fig. 2:**
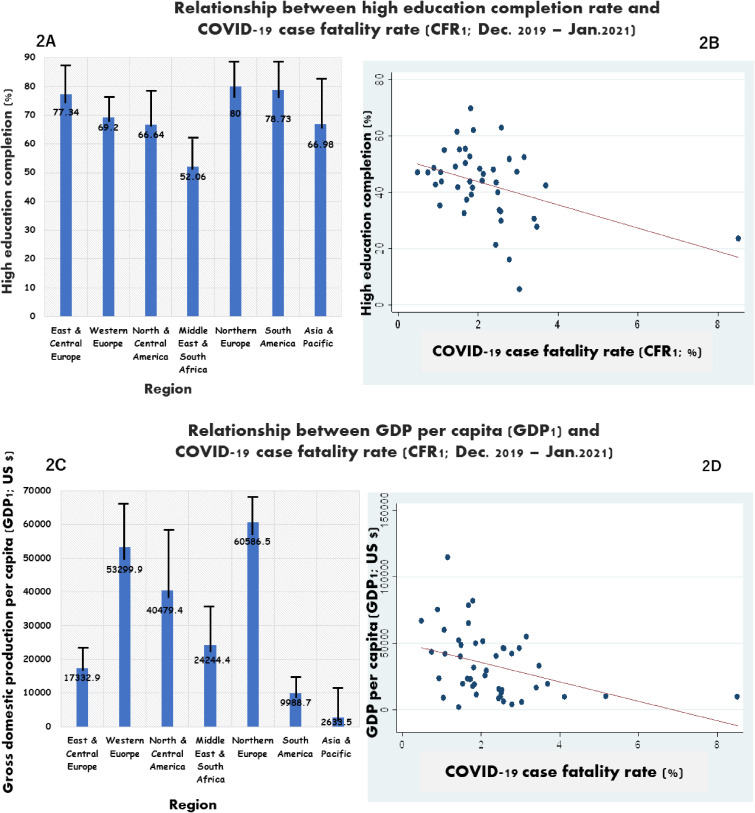
High education completion rate (A), GDP (C), their relationship with CFR (B, D) in first year of pandemic CFR, case fatality rate; GDP, gross domestic product. Figure 2 shows the higher the proportion or rate of population with completion of tertiary education, the lower the disease fatality rate (A, B); similarly, the higher country’s GDP per capita gets, the lower gets is the trend of COVID-19 related mortality (C, D).

At regional level, data related to the first year of the pandemic showed that highest CFR1 was observed for north & central America region, 4.25 (3.71) %, followed by South America (2.5 (0.1) %), Asia & Pacific region (2.41 (1.43) %) and East & central Europe (2.24 (0.98) %), whereas lowest CFR2 was observed in northern Europe, 1.19 (0.6) % (Fig. 1A–B). Figure 2A–B shows the distribution high education completion rate and its linear relationship with COVID-19 case fatality rate (CFR1). Northern Europe was the region with highest high education completion rate (80 (7.4) %) followed by South America (78.7 (20.3) %) and East & central Europe (77.3 (26.9) %); the lowest rate was found in the Middle-east region/South Africa (Fig. 2A). A linear relationship was observed between country’s population rate with tertiary education and CFR1, and CFR1 tended to decrease when the high education completion rate increased (Fig. 2B). Considering economic aspect, northern Europe had also the highest mean GDP *per capita* (GDP1: 60,586.5 (10,959.4) US$) and Asia-Pacific region had the lowest one (2,633.5 (2,0957.7) US$) (Fig. 2C). A linear relationship was observed between GDP1 and CFR1 (Fig. 2D).

**Fig. 3:**
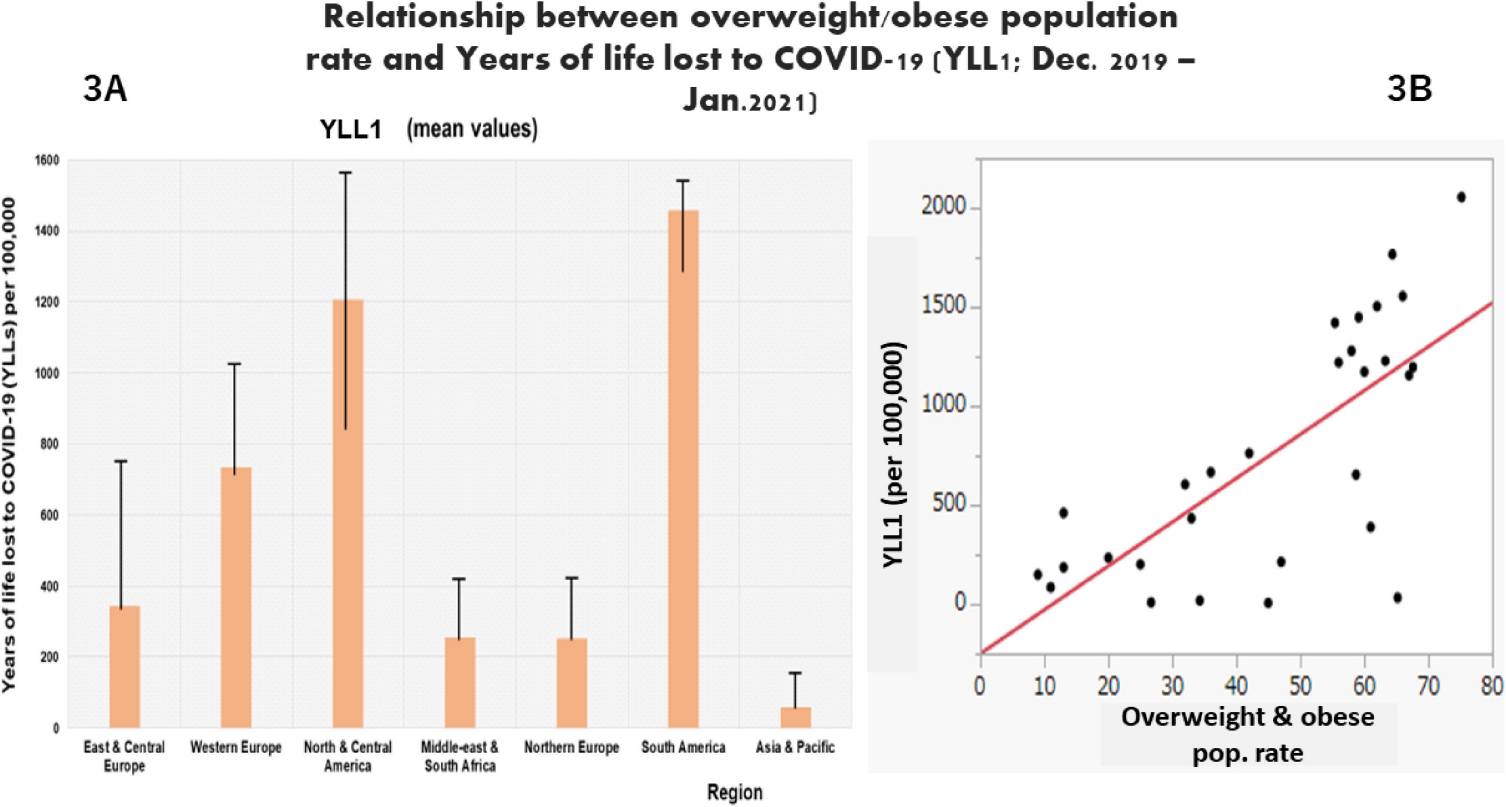
COVID-19 YLL (A) and its relationship with overweight/obese population rate (B) in first year of pandemic YLL1, years of life lost due to the disease (per 100,000 people) in the first year of the pandemic. The figure 3 shows a higher mean YLL in North & central America region, followed by South America, whereas the lowest YLLs was observed in Asia & Pacific region (A); on the other hand, the figure shows an increasing trend of YLLs when the rate of overweight and obese population increases (B).

Figure 3A–B shows YLL1 (per 100,000) trend according to region, and its relationship with overweight/obese population rate. Highest YLL1 was observed in South America, 1457.5 (274.8), followed by north & central America, 1207.3 (908.1) per 100,000; whereas Asia & Pacific region had the lowest YLL1, 57.57 (76.5) (p < 0.001) (Fig. 3A–B).

### 3.2. Association between outcome variables (CFR1, YLLs1) and covariates in first year of the pandemic

Table 3 shows results of the analysis for data related to the first year of the pandemic, with adjustment for country’s elderly population rate. CFR1 was inversely associated with GDP *per capita* [β = −6.5 (2.5); 95%CI: −11.6–1.36; p < 0.05] and higher education completion rate [β = −3.05 (1.47); 95%CI: −6.04–(−0.06); p < 0.05]. On the other hand, YLL1 was positively associated with overweight and obese population rate [β = 19.7 (16.3); 95%CI: 6.14–55.65; p < 0.05] and elderly population rate [β = 18.6 (16.6); 95%CI: 12.13–34.9; p < 0.05].

**Table 3:** Relationship between outcome variables and covariates with adjustment for elderly population rate (Dec. 2019–Jan. 2021)

**Covariates**	**CFR1**			**YLL1**		
		
	Beta (SE)	95% CI	p	Beta (SE)	95% CI	p
		
High education completion rate	−3.05 (1.47)	−6.04–(−0.06)	**0.045**	−0.03 (0.00)	−0.01–0.01	0.907
GDP per capita (GDP1)	−6.5 (2.5)	−11.6–1.36	**0.014**	−3.4 (6.6)	−16.7–9.8	0.603
Democracy index	−1.3 (0.2)	−0.63–1.02	0.071	−206.9 (91.6)	−392.2–(−21.5)	0.080
Nurse per 10,000	−1.04 (0.5)	−1.98–(−0.09)	0.053	−0.01 (0.1)	−0.003–0.003	0.341
Doctor per 10,000	−0.09 (0.1)	−0.29–0.09	0.318	0.01 (0.0)	−0.003–0.005	0.645
Elderly population rate (≥65 y.)	0.7 (0.6)	1.89–0.53	0.264	18.6 (16.6)	12.13–34.9	**0.039**
Overweight/obese pop. rate	0.2 (0.1)	0.11–0.31	0.264	19.7 (16.3)	6.14–55.65	**0.025**

**Notes:** CFR, case fatality rate; YLL, years of life lost to COVID-19; CI, confidence interval; p, p-value; SE, standard error.

### 3.3. Regional distribution of outcome variables (CFR2, YLL2) and their determinants for the 2-year study period (Dec. 2019–31 Dec. 2021)

**Fig. 4:**
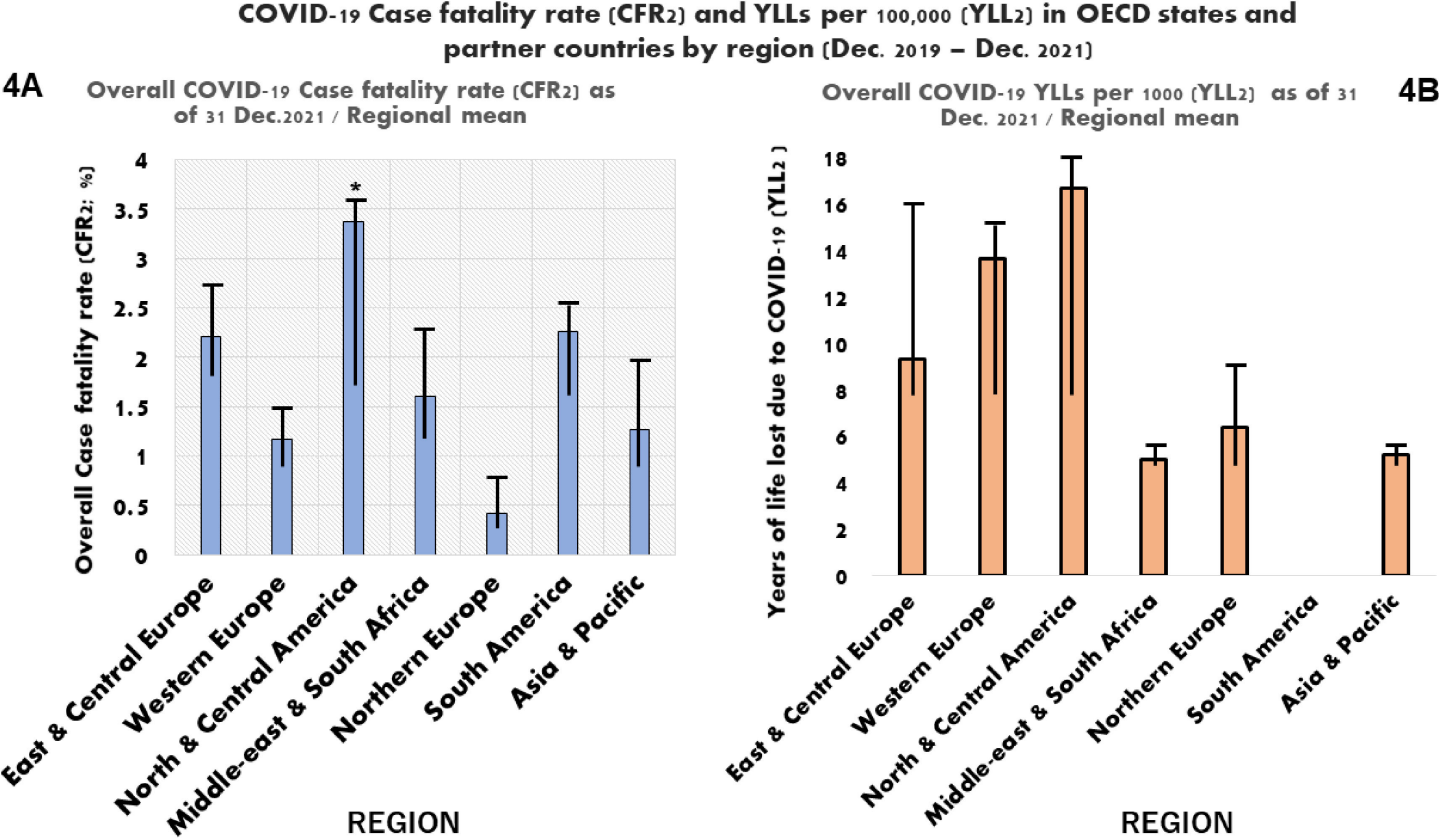
Regional distribution of COVID-19 CFR and YLL as of 31 December 2021 CFR2, COVID-19 case fatality rate as of 31 December 2021; YLL2, years of life lost due to COVID-19 as of 31 December 2021; *, p-value less than 0.05.

Overall regional mean CFR (CFR2) was highest for North & central America region, 3.37 (3.19) %, whereas northern Europe had the lowest one, 0.42 (0.44) % (p < 0.05). Highest YLLs (YLL2) was observed in North & central America, 16.7 (13) per 1,000; followed by western Europe, 13.65 (8.51) per 1,000; whereas lowest YLLs was observed in middle-east & south Africa, 5 (0.1) per 1,000 (Fig. 4A–B).

As shown in Table 4, inverse correlations were observed between country-level CFR and high education completion rate (r = −0.36; p < 0.05), rate of nurse per 1000 (r = −0.48; p < 0.05) and GDP *per capita* (r = −0.54; p < 0.001), whereas YLL was positively correlated with elderly population rate (r = 0.66; p < 0.05) and overweight/obese population rate (r = 0.55; p < 0.05) (Table 4).

**Table 4:** Relationship between outcome variables and covariates (31 Dec. 2019–31 Dec. 2021)

**Variable**	**CFR2 ** **(31 Dec. 2021)**		**YLL2/1000 ** **(31 Dec. 2021)**	
		
	R	p trend	R	p trend
		
High education completion (%)	−0.36	**0.017**	−0.25	0.242
Elderly population rate	−0.29	0.052	0.66	**0.034**
Overweight/obese population (%)	0.31	0.084	0.55	**0.047**
Doctors per 10,000	−0.25	0.166	−0.27	0.224
Nurses per 10,000	−0.48	**0.022**	0.02	0.992
GDP *per capita*	−0.54	**0.000**	−0.25	0.248
Democracy index	−0.28	0.059	−0.29	0.15

*Notes: CFR2, case fatality rate for the period between 31 December 2019 to 31 December 2021; YLL2, years of life lost due to COVID-19 between 31 Dec. 2019 to 31 Dec. 2021.

It was observed that countries with high education completion rate, higher rate of nurse per 1000 population and high GDP *per capita* tended to have relatively lower CFR; whereas those with high elderly population and high overweight and obese population rates were likely to have higher YLLs (Suppl. material 1A–B–C–D–E–F).

## Discussion

This paper explored the burden of SARS-CoV-2 infection from the start of the outbreak in China in 2019 through December 2021 at country and regional levels, and factors associated with COVID-19 CFR and YLL across 48 OECD member states and partner countries. This study showed that north & central America region had the highest COVID-19-related CFR in the first year of the pandemic as compared with other regions, with the USA being the top country with highest disease mortality. This trend did not change throughout the study period. Regarding YLLs, south America was the region that bore the heaviest burden in the first year of the pandemic; but England and the US were countries with highest YLLs when considering the 2-year study period.

Additionally, it was observed that GDP *per capita* and higher education completion rate were inversely associated with CFR both in the first year of the pandemic and through the 2-year study period, suggesting that OECD member and partner countries with high income and high education level tended to have low COVID-19-related fatality. These findings are in line with observations from previous studies conducted in France and the United States, showing that economically disadvantaged populations living in deprived areas bear the heaviest COVID-19 burden [24, 25]. A study conducted by Paakkari and Okan [26] has shown that education, especially health literacy, can be considered as a crucial tool in disease prevention, and populations with low literacy have been reported to be less responsive to health education and less likely to use disease preventive measures, as compared with literate ones [27]. Given that education can improve health literacy, there is a possibility high education attainment could influence adherence to anti-COVID-19 public health or preventive measures and reduce disease morbidity and mortality.

Furthermore, when considering the entire 2-year period of our study, positive relationship was observed between years of life lost to COVID-19 and overweight/obese population and also elderly population rates. This suggests that countries with high rate of overweight and/or obese population, as well as those with high proportion of aging population are more likely to have high mortality due to SARS-CoV-2 infection.

In fact, it has been reported that noncommunicable diseases (NCDs), including metabolic disorders such as hypercholesterolemia (LDL-c), obesity, metabolic syndrome and diabetes, are high-risk factors for severe SARS-CoV-2 infection and mortality. A global systematic review and meta-analysis that included 76 studies showed a 2.57 (odds ratio range: 1.31–5.05) higher risk of severe COVID-19 in patients with severe obesity [28]. Additionally, two recent COVID-19 ecological studies [14, 29] showed that countries with high proportion of people aged 80 years or older and those with high obese population rates tended to have high COVID-19 mortality rates. Thus, countries with aging population issue and those with high rates of population at risk or with metabolic disease are more likely to experience more severe COVID-19 outcomes. This fact corroborates with findings from our study that showed a disproportionate impact of the pandemic among OECD countries, in terms of disease mortality.

Our study also showed a strong association between YLLs and elderly population rate. A meta-analysis that included 13 studies has shown a 6-fold high risk of COVID-19 related death in patients over 65 years of age as compared to younger subjects [30]. The fact that chronic conditions such as lifestyle-related NCDs are quite common among elderly people may also explain this fining. That is because NCDs have been associated to severe COVID-19 outcomes. For example, in our study, both elderly population rate and overweight/obese population rate -which is one of metabolic risk factor- were both independently associated with COVID-19 fatality.

Furthermore, our study showed an inverse relationship between the ratio nurse per 10,000 and COVID-19 fatalities, suggesting that OECD states and partner countries with low ratio tended to have more COVID-19 fatalities. Another recent ecological study that searched to determine the predictors of SARS-CoV-2 infection also showed that the ratio nurses/midwives per population was inversely associated with COVID-19 mortality [31]. Nurses are among frontliners to safeguard the lives of affected persons during health crises. Obviously, the health systems of many countries are getting overwhelmed by COVID-19 pandemic. Thus, countries facing a critical shortage of nurses could be more likely to suffer severe health outcomes in COVID-19 patients. A recent Indonesian study has reported about the human resources challenge that the country’s health system has been struggling with, as the country was facing a significant shortage of nurses and midwives, suggesting that effective pandemic response would require governments to increase the healthcare workforce and capacity [32].

Our study is one the rare ones that combined a variety of reliable data sources in a single analysis, showing the trend of COVID-12 burden at country and regional levels. It revealed that old age and overweight/obesity represent the key determinants of COVID-19 burden and fatality among OECD states and partner countries in the first two years of the pandemic. These health determinants of COVID-19 can be improved through the promotion of heathy longevity and the optimization of health programs oriented toward the prevention of lifestyle-related NCDs.

Nonetheless, this study has a number of limitations. Findings derive from databases where individual country’s data for selected variables are gathered. They apply to the group of 48 countries studied as whole but not to one particular nation. Some countries among the OECD members and partners may experience greater burden from COVID-19 pandemic than others. With regard to socioeconomic factors and health indicators, there are noticeable disparities according to regions. Thus, regional comparisons would have provided different epidemiologic features, particularly in terms of COVID-19-associated risk factors. Additionally, there was lack of data related to health system indicators (doctor per 10,000 population, nurse per 10,000 population) for two countries from the south America region. This could somewhat influence the result in terms of relationship between ratio of the above-mentioned health system indicator and outcome variables. Thus, the related finding should be interpreted cautiously.

As a conclusion, this study is the first to provide a comprehensive analysis of COVID-19 burden across OECD members states and partner countries. It has highlighted the epidemiologic trend of COVID-19 burden at country and regional levels. More importantly, economic status at individual level (GDP *per capita*), old age and lifestyle-related disorders such as obesity were found to be major social and health determinants of COVID-19 burden among OECD countries. These findings suggest that a robust economy, interventions designed to promote healthy longevity and prevent weight gain in at-risk individuals might reduce COVID-19 burden and fatality among OECD states and partner countries.

## Supplementary information

**Additional file 1:** **S-1A** Relationship between Higher education completion rate and COVID-19 Case fatality rate (CFR2; Dec. 2019 to Dec. 2021). **S-1B** Relationship between GDP per capita (GDP2) and COVID-19 case fatality rate (CFR2; 31 Dec. 2019 to 31 Dec. 2021). **S-1C** Relationship between country’s democracy index and COVID-19 Case fatality rate (CFR2; 31 Dec. 2019 to 31 Dec. 2021).

**Additional file 2:** **S-1D** Relationship between country’s Nurse per 1000 ratio and COVID-19 YLLs per 1000 (YLL2; 31 Dec. 2019 to 31 Dec. 2021). **S-1E** Relationship between country’s Elderly population rate and COVID-19 YLLs per 1000 (YLL2; 31 Dec. 2019 to 31 Dec. 2021). **S-1F** Relationship between country’s overweight/obese pop. rate and COVID-19 YLLs/1000 (YLL2; 31 Dec. 2019 to 31 Dec. 2021).

**Additional file 3:** **Supplementary material 2.** Statistical analysis for multicollinearity of the variables.

## Declaration

### Availability of data and materials

The datasets used in this study can be obtained from the corresponding authors.
