# Supplementary figures and images for: Country-level and regional COVID-19 burden and determinants across OECD member states and partner countries
Source: Environ Health Prev Med. 2022 Oct 21;27:41. doi: 10.1265/ehpm.22-00054 (PMC9640741; doi:10.1265/ehpm.22-00054)

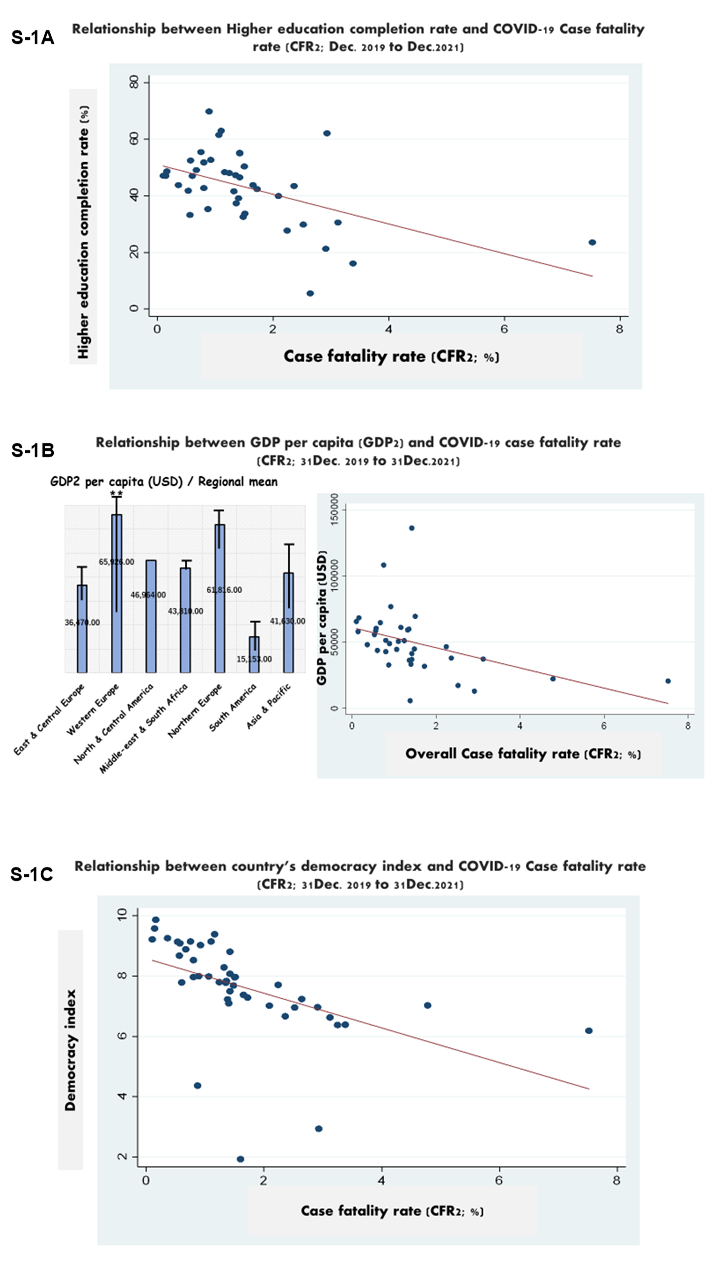

Supplement: Supplementary file 1 — Additional file 1: S-1A Relationship between Higher education completion rate and COVID-19 Case fatality rate (CFR2; Dec. 2019 to Dec. 2021). S-1B Relationship between GDP per capita (GDP2) and COVID-19 case fatality rate (CFR2; 31 Dec. 2019 to 31 Dec. 2021). S-1C Relationship between country’s democracy index and COVID-19 Case fatality rate (CFR2; 31 Dec. 2019 to 31 Dec. 2021). [file ehpm-27-041-s001.tif]

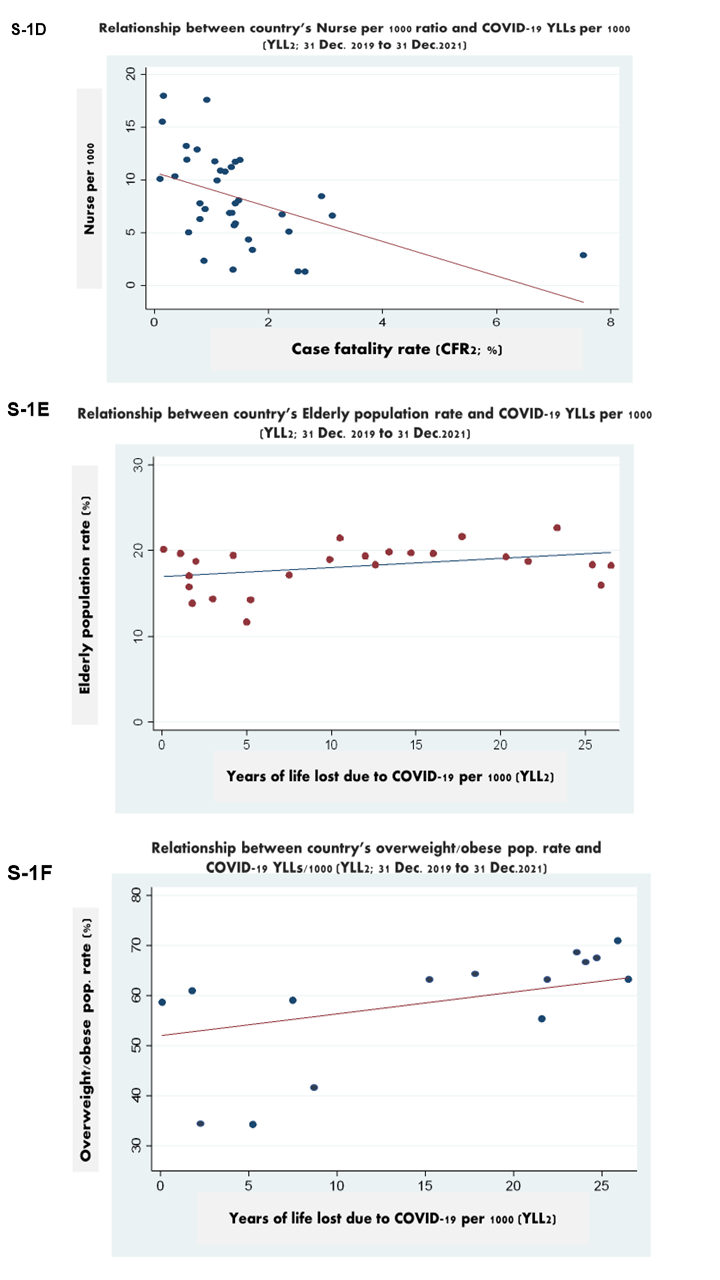

Supplement: Supplementary file 2 — Additional file 2: S-1D Relationship between country’s Nurse per 1000 ratio and COVID-19 YLLs per 1000 (YLL2; 31 Dec. 2019 to 31 Dec. 2021). S-1E Relationship between country’s Elderly population rate and COVID-19 YLLs per 1000 (YLL2; 31 Dec. 2019 to 31 Dec. 2021). S-1F Relationship between country’s overweight/obese pop. rate and COVID-19 YLLs/1000 (YLL2; 31 Dec. 2019 to 31 Dec. 2021). [file ehpm-27-041-s002.tif]
