# Supplementary material for: Country-level and regional COVID-19 burden and determinants across OECD member states and partner countries
Source: Environ Health Prev Med. 2022 Oct 21;27:41. doi: 10.1265/ehpm.22-00054 (PMC9640741; doi:10.1265/ehpm.22-00054)
Supplement: Supplementary file 3 — Additional file 3: Supplementary material 2. Statistical analysis for multicollinearity of the variables. [file ehpm-27-041-s003.docx]

**Supplementary material 2.** Statistical analysis for multicollinearity of the variables

Checking for Multicollinearity:

**. vif**

Variable | VIF 1/VIF

-------------+----------------------

overweight~n | 3.44 0.225430

population~n | 3.58 0.279389

elderlypop~n | 2.84 0.352032

nursesp~1000 | 2.58 0.387817

doctors~1000 | 1.89 0.528007

-------------+----------------------

Mean VIF | 3.07

As a result of vif test using Stata software, given the low VIF values (below 5), no collinearity observed between the selected covariates.
